# Empirical development and verification of career well-being scale for teachers in Taiwan: Implications for workplace counseling

**DOI:** 10.3389/fpsyg.2022.855286

**Published:** 2022-12-14

**Authors:** Min-Ning Yu, Peter Yang

**Affiliations:** ^1^Department of Education, National Chengchi University, Taipei City, Taiwan; ^2^Department of Counseling, National Chiayi University, Chiayi City, Taiwan

**Keywords:** basic psychological needs, career agency, career counseling, career growth needs, proactive career behavior, psychological well-being, subjective well-being, workplace counseling

## Abstract

As it is one decade since the establishment of Kidd’s model, an analysis of the career well-being (CWB) experienced by Eastern workers is both timely and necessary. To this end, we conducted a series of logical investigations of CWB in Taiwanese school teachers. Study 1 was conducted to conceptualize the main features of CWB (*n* = 135), and Study 2 was conducted using exploratory factor analysis to determine the validity of a four-factor measurement structure (*n* = 191). In Study 3, tests were completed to confirm the factor structure of the CWB (*n* = 533). Accordingly, we established a theory-based CWB measurement approach, and statistical analysis verified the convergent, divergent, and criterion validity of our CWB measurement model. Exploratory structural equation modeling rather than confirmatory factor analysis is recommended in discussions of CWB theory and practice in educational contexts. However, because our sample solely comprised Taiwanese teachers, our results are not generalizable to other occupations or cultures, even Eastern or Chinese-derived cultures. Implications for both theory and workplace counseling practice are presented.

## Introduction

### Career well-being model

Kidd (2008) highlighted the necessity of analyzing the features of career well-being (CWB). Distinct from common conceptualizations of well-being (e.g., Ryff, 1989; Diener, 2000), Kidd focused first on emotions (Kidd, 1998, 2004) and subsequently on the general sense of wellness experienced during one’s working life (i.e., *the feeling of a career going well*; Kidd, 2008, p. 175). Kidd (2008) revealed that among British workers, feelings of CWB ranged broadly, influencing both work and non-work life. Based on the results of this qualitative study, several sources of crucial career experiences were recognized in terms of their associations with the positive and negative effects, resulting in the establishment of a CWB model.

When we examined the theoretical perspective used to establish the CWB model, we discovered that Kidd (2008) attempted to maintain a balance in the treatment of “hedonic” and “eudaimonic” well-being (Proctor et al., 2015). The positive and negative effects conceptualized in Diener’s (2000) analysis of subjective well-being (SWB) were employed by Kidd (2008) to investigate the facilitators and threats of CWB. Compared with the psychological well-being (PWB) model of Ryff and Keyes (1995), interpersonal relationships (positive relations with others), relationship with the organization (autonomy, environmental mastery), work performance (self-acceptance), sense of purpose (purpose in life), and learning and development (personal growth) were also analyzed in the CWB model (PWB terms are parenthetically referenced). However, career transitions were found to be an additional critical component, indicating that CWB might depend on developing into a new role, career pattern, or organization. Furthermore, Kidd (2008) demonstrated that both the work dimension and life dimension of a career (non-work) substantially influenced the sense of CWB; for example, work–life issues (e.g., work–home life balance) and living pattern changes (e.g., learning a hobby or studying a religion) were both reported. These results revealed that although career perspectives are related to the work dimension, a deeper understanding of workers’ well-being experienced throughout their lives and in their livelihood career choices and development requires that the work–non-work interface (work–family conflict, enrichment, and balance; Geurts and Demerouti, 2003) be included in any CWB study.

Studies on CWB have revealed different features of affective experience in relation to careers. In contrast to the emotions that accompany particular events at a given time and are usually temporary, long-term well-being is the key concern of career studies regarding career events and processes.

Although a preliminary CWB framework was established by Kidd (2008), no systematic follow-up studies have been conducted (Hartung, 2011). Recent studies have examined emotions’ role in career development (e.g., job searches and novice job seekers’ decision-making process; Bonaccio et al., 2014). However, the emotions perceived at a given time do not necessarily lead to an overall perception of wellness in a career; this discrepancy necessitates analysis of how workers evaluate their CWB. Few studies (e.g., Chen and Haller, 2015) have discussed the advancement of CWB theory.

Theoretical and methodological analyses of CWB are largely absent in the literature, and the majority of CWB studies have been conducted in Western societies. Few investigations of CWB have been undertaken in Eastern cultures. As a decade has passed since Kidd’s (2008) model, a timely investigation of the CWB of Eastern workers is necessary.

### Chinese-context career well-being model development rationale

Numerous investigations have been conducted into culturally Chinese workers’ subjective and psychological well-being in mainland China, Hong Kong, Singapore, and Taiwan. Among these investigations, Tan et al. (2009), which focused on Singapore, was heavily influenced by the World Values Survey initiated in 1981 and concentrated on the topics of quality of life and well-being. They also analyzed results from the Asia Barometer Survey and highlighted how to strengthen social capital and democracy in Chinese societies. This approach of emphasizing the implications of SWB and happiness studies for policy makers as well as for community development to promote and advocate quality of life is actually a similar approach to using the CUHK Hong Kong Quality of Life Index that Chan et al. (2005) established near the same time to track Chinese quality of life, which they conceptualized and measured in terms of health, social, cultural, leisure, economic, and environmental aspects.

In Taiwan, apart from the heavy emphasis, as in Hong Kong and Singapore, on measuring national well-being to promote economic growth and social cohesion—as a governance policy (OECD, 2011), several series of investigations into well-being have been conducted since the 1990s. Lu and colleagues examined the relationship between SWB and personality traits (e.g., extroversion, neuroticism) and psychosocial variables (e.g., social support), and their findings demonstrated that cultural variations should be taken into account in the study of happiness (Argyle and Lu, 1990; Lu and Argyle, 1992; Lu, 1995). Approximately one decade prior, Yu and colleagues concentrated on primary and high school teachers’ SWB, with a mental health model established to explain the feature of flourishing teachers (Yu et al., 2011, 2018; Yu, 2021).

More recently, a trend in research has been to examine various aspects of well-being in mainland China, leading to an accumulation of findings explaining the cultural differences between Western and Eastern societies. Churchill and Mishra (2017), for example, found that the effects of social capital (trust and social networks) on well-being in China were relatively weaker than the effect of income. This differed from the Western view that social capital is a stronger determinant of well-being than income. In addition to the possible variations that appear in the associations among Chinese SWB and its determinants and outcomes, the fundamental issues pertain to conceptual and measurement problems that may restrict the theoretical advancements of SWB research in China. Just as Chen and Davey (2008) highlighted much earlier, theoretical and methodological advancements should be made to make sense of which methods can actually help answer which type of research questions. They reviewed nearly 800 studies published in Chinese language journals since 1999 and found several problems, including wide deployment of amended versions of Western scales as the main measurement instrument, overuse of student samples, and methodological weaknesses. The key terms in the literature, such as happiness and SWB, should not been assumed to be universal across cultures. They may convey different meanings, particularly in China and the Chinese language (Davey and Rato, 2012).

Despite the aforementioned perspectives that have been formulated and discussed in Chinese societies, CWB perceptions among Chinese workers is a research gap. A study focused on the general sense of wellness that they experience over the course of their working life should be conducted to determine the characteristics of CWB in this particular cultural context. Although Kidd’s (2008) pioneering work was set in the West and provides a preliminary conceptualization of CWB, cultural variance may influence culturally Chinese workers’ general sense of wellness as well as their personal feelings of pleasure that last throughout their career development. Apart from Chen and Haller (2015), theoretical and methodological analyses of CWB in Chinese societies are currently lacking. This study aims to fill that gap.

### Conceptual and measurement concerns related to theoretical advancements

Despite considerable progress that has been made in well-being research, several conceptual and measurement concerns have restricted the advancement of CWB theory.

First, an appropriate definition and conceptualization of contemporary workers’ well-being from their personal perspectives vis-à-vis long-term working life experiences are necessary. The definition of CWB has been established from a long-term perspective (Kidd, 2008) rather than from a short-term perspective, with an aim to understand how employees’ work experiences relate to how they evaluate subjective feelings of success, wellness, and quality of life. Accordingly, workers’ relationship with work as well as how their career identity is constructed in the progression of their working life can be examined (Coupland, 2004). Corresponding to this emphasis on careers over long periods of time, an overall evaluation of well-being, rather than positive emotions at one point in time, is more appropriate for evaluating workers’ wellness because this approach analyzes how workers feel about the sequence of jobs they undertake over the course of their working lifetime.

Second, both quantitative and qualitative analyses should be undertaken to examine the phenomena that occur across different research phases. Methodologically, using a qualitative approach to explore the fundamental components of CWB within Eastern cultures is typically appropriate at the early phase of the study. After the establishment of a clear conceptualization, it is necessary to establish a psychometrically sound measure to evaluate Eastern workers’ CWB. Moreover, a quantitative approach can confirm the conceptualization that has been previously established using a qualitative approach. In addition to exploratory factor analysis (EFA), structural equation modeling (SEM) can be used to verify factorial structures; employing empirical investigations to guide model modifications can lead to a deeper understanding of the theoretical implications of CWB in different cultures. To enable a broader understanding of the organization of CWB (Fresno et al., 2020) and prevent misuse of fit indices due to unethical and questionable model modifications (Stone, 2021), exploratory SEM (ESEM) rather than typical confirmatory factor analysis (CFA) can be used as a means of verifying the factor structure of a set of observed variables. Doing so can provide useful information for determining the latent optimal structure of CWB established in the literature (Asparouhov and Muthén, 2009). CFA has been criticized for its overly simplistic, restrictive, and idealistic independent cluster model assumption (Xiao et al., 2019; Dierendonck et al., 2021), which has led to CFA frequently failing to meet several standards of good measurement (e.g., goodness-of-fit, measurement invariance, and well-differentiated factors; Marsh et al., 2020). Because of this, ESEM has been proposed as a potential alternative. ESEM overcomes the aforementioned challenges by incorporating the most favorable elements of EFA and CFA (Van Zyl and Ten Klooster, 2021). ESEM uses simulated data with cross-loadings and has increasingly been recognized as superior to CFA (e.g., Xiao et al., 2019). For these reasons, EFA, CFA, and ESEM can be used to establish a psychometrically sound measure for evaluating Eastern workers’ CWB.

Third, to support CWB conceptualization, we must examine the relationships of CWB with career outcomes as well as with work outcomes. Without evidence for divergent and convergent validity, the characteristics of CWB cannot be clarified and future studies would not be able to provide a solid foundation for the most appropriate use of this concept. As CWB studies in the literature have been limited to theoretical investigations, the provision of empirical evidence can help explain the relationships of CWB with additional work variables (e.g., work-related self-efficacy, perceived work-based social support), leading to advancements in theory and practice in this area. Before examining different mechanisms involved in the process of causal explanations (e.g., career outcomes), a cross-sectional analysis should be undertaken to demonstrate the relationship between CWB and critical work variables to ensure convergent, divergent, and criterion validity.

Fourth, it is crucial to establish a concise method for measuring CWB, as recommended by Cheng and Chan (2005). A short version of the CWB scale, with robust methods of psychological analysis, would contribute to the utility of CWB for employee selection, training, and workplace counseling. After reviewing the methods of measuring well-being reported in the literature, Cheng and Chan (2005) argued that four items for each component are sufficient for the measurement of well-being. However, for an accurate measurement of well-being, the method employed should have a firm theoretical basis (Clark and Watson, 1995). Hence, researchers should measure CWB using methods that are both theoretically and psychometrically sound.

Finally, to advance the theory of CWB, self-determination theory (SDT; Ryan and Deci, 2000) and career self-determination theory (CSDT; Chen, 2017), which is more specified in career studies, can be employed to explain the mechanism underlying why workers are motivated to pursue a more satisfying and favorable career and thus provide a conceptualization on which to base theoretical CWB measurement. Inspired by SDT emphasizing inner human strength and the potential to enhance well-being in life (Ryan and Deci, 2000), Chen (2017) argued that CWB is initiated and strengthened by the satisfaction of three fundamental psychological needs in career processes: namely, career autonomy, career competence, and career relatedness. This meta-theory (i.e., CSDT) established by Chen (2017) provides theoretical integration of existing constructs, which are associated with the overall perception of wellness in a career. Self-concept, vocational interest, meanings, and meaning-making are essential to career autonomy, whereas career competence is shaped by competence essentials (given capacities and gained skills), learning (intentional learning and causal learning), and self-efficacy. Career relatedness is grounded in the context where primary reference points are given for human perception (e.g., CWB) to understand career events, make sense of career intentions, and take career actions. Contextual meaning-making associated with the search for CWB in career experiences is mainly determined by the workers’ essential relatedness to significant others (e.g., parents and family members) as well as their general relatedness to those people who are important to them (e.g., supervisor, coworkers).

The needs of competence, relatedness, and autonomy are the basic psychological needs related to the pursuit of well-being (Ryan and Deci, 2000). They relate to workers’ personal feelings of pleasure that last throughout their career development. Clarifying “agency” can help explain why and how workers experience affective perceptions differently in a given career event, during the sequence of jobs they have over the course of their working life, and even when doing similar jobs. Accordingly, CWB can be examined with respect to intentions and psychological needs. Integrating SDT into the study of CWB to highlight the crucial role of needs fulfillment in optimal psychological functioning, as Chen (2017) recommended, helps to respond to the current lack of theoretical grounding for the different features of affective experience in relation to careers (Hartung, 2011) and facilitates the establishment of a theory-based CWB measurement approach.

### Purpose of the current study

We defined CWB as the wellness experienced throughout one’s careers. Three studies were conducted in Taiwan, a non-Western culture, to examine the characteristics of CWB.

The research questions were

Study 1: What would be the main features of CWB in Taiwan?

Study 2: Would the conceptualization of CWB established in Study 1 be supported statistically by the measurement properties? Could EFA be applied for this multidimensional construct, and could the convergent, divergent, and criterion validity of this CWB measure be established?

Study 3: Would the factor structure of the modified CWB measure of Study 2 be supported statistically when CFA and ESEM were used?

Cheng and Chan (2005) argued that the well-being model is not equally applicable to all occupations. Therefore, we explored the CWB experienced by workers in a single occupation: school teacher. The findings obtained in this series of investigations of Taiwanese school teachers’ CWB may offer a direction for future studies seeking to identify variations in CWB among occupations. However, because the sample solely comprises Taiwanese teachers, determining whether the differences in our findings and those of other CWB studies resulted from differences in Eastern and Western cultures or in teachers and those working in other professions is impossible.

## Study 1: Conceptualization to explain the features of career well-being in Taiwan

Study 1 was conducted to conceptualize CWB. To develop a theory-based CWB measurement approach, the results obtained in this qualitative study were further analyzed by applying SDT; specifically, in light of the argument of Deci and Ryan (2000) regarding the crucial role of needs fulfillment in optimal psychological functioning.

### Method

#### Participants and research instrument

An open-ended questionnaire was established to collect school teachers’ CWB experiences in Taiwan. By adapting Kidd’s (2008) interpretation, CWB was defined as “a sense of wellness in that teachers feel positive about their teaching careers.” CWB was analyzed by asking teachers the following question: “Can you identify a time, preferably within the last 3 years, when you felt particularly positive about your teaching career?” The participants were asked to recall the sequence of jobs they had over the course of their working life and then to identify a period of time in which they felt particularly positive about their career (Kidd, 2008, p. 171).

#### Procedure

To acquire information on a diverse range of career experiences, the participants were enrolled from a continuing education program for enhancing current teachers’ skills in school guidance and counseling. The participants worked at schools in both major cities and rural areas. After completing the program, some teachers volunteered to participate in this study. A take-home survey was administered (272 copies), and 158 copies were returned (response rate of 58.09%). In return for their time and efforts, the participants were given feedback in the form of research findings and were also entered into a raffle for several USB flash drives. After excluding 23 surveys with incomplete information, 135 valid copies were obtained. The participants represented a diverse group: 108 were women, 75 had a master’s degree or doctorate, and the age range was 25–51 years.

Regarding the methodology, a qualitative content analysis (Creswell and Poth, 2017) involving four steps was employed. In the first step, a coding scheme was devised from 30 survey responses to code the remaining participants’ responses. In the second step, the responses that could not be categorized into this preliminary framework were discussed, followed by the establishment of an extended edition of the original categorization approach (i.e., a revised coding scheme) to code all the responses again. The procedure was repeated until there were no responses that could not be categorized. In the third step, to strengthen the validity of the results, we cross-checked 70 copies that were coded independently by two external auditors who used the final coding scheme established in the second step (Creswell and Poth, 2017). This step was conducted to detect conflicting categorizations to ensure procedural rigor and improve the credibility of the qualitative inquiry. An acceptable level of interrater reliability (59/70, 84.29%) was obtained, but we engaged in continuous consultations and discussed divergent opinions concerning the categorizations. This procedure to improve the consistency among coders was crucial for cross-checking the categorization results. Feedback that disagreed with the results provided opportunities for further discussion. Subsequently, in the final step, by resolving all conflicting categorizations, we established a credible description of CWB components.

### Results and discussion

#### Preliminary categorization

A categorization of CWB was established for grouping career experiences that created the perception of wellness throughout careers. Eight CWB components are listed in Table 1, along with examples and the frequency with which these components were reported by participants. A wide range of wellness was exhibited for the multidimensional components throughout the teaching careers.

**TABLE 1:** Eight CWB components.

CWB components	Categorizations		Frequencies
	Positive career experiences	Examples	
Component 1: Self-development and learning	Professional development in education Obtained a growth opportunity through advancement in a current job Participated in on-the-job (OJT) training and development programs Learned knowledge or skills through a program of study Received a diploma (a master’s or doctoral degree)	Pursued a part-time postgraduate course (P31); learned how to apply the flipped classroom model for students by attending a workshop (P60)	19
Component 2: Work performance and achievement	Performing to the best of one’s ability Personal interests in line with scheduled tasks Got along with students Felt a sense of achievement in maintaining positive relationships with parents Had a clear target or direction for continued advancements in work performance	Worked with students to accomplish satisfactory graduation performance (P4); Successfully communicated with the parents of a student with attention-deficit/hyperactivity disorder (P79)	62
Component 3: Interpersonal relationships and support at work	Worked in a friendly workplace Got along with colleagues Got along with the school supervisor or administrative staff Received support or feedback from others Received recognition from others	Had colleagues who recognized and encouraged me (P22); supported each other in the school (P101)	21
Component 4: Career transition and changes	Held a new position Established a new career pattern Expanded one’s social network and living community due to a new job	Moved to the current school after a decade in the comfort zone (previous school) (P54); Taught overseas and had fresh experiences in Ho Chi Minh City (P123)	11
Component 5: A sense of purpose and direction for the future	Had a clear target or direction for the future Pursued clear targets for achieving a goal Saw prospects for future development	Received insights from the long-term administrative job that was associated with a feeling of exhaustion (P77); had personal goals regarding how to evoke feelings of love from students (P101)	12
Component 6: Work–life balance	Home life or leisure time not affected by work Achieved a balance between work and family life	Met a Buddhist master and initiated Buddhist studies (P34); had sufficient time for family members and to maintain a good quality of life (P88)	4
Component 7: Autonomy at work	Perceived autonomy at work	Managed the class in my own way (P65); what I did for students was really what I intended rather than an administrative mandate (P2)	4
Component 8: Participation in decision-making	Participated in important decisions	Planned an off-campus activity with the administrative team (P93); Performed an administrative role (P134)	2

*n* = 135; P refers to participant.

“Self-development and learning,” “work performance and achievement,” and “interpersonal relationships and support at work” were the most frequently mentioned experiences that participants reported in perceiving that their careers were going well. Among these components, “work performance and achievements” was noted in 45.93% of the participants’ statements (62/135). A large proportion of participants reported that “interpersonal relationships and support at work” was a crucial component that elicited the feeling of a career going well (21/135, 15.56%). Of note, “self-development and learning” was reported as crucial by 19 respondents (14.07%), which was a slightly higher proportion of participants than that in Kidd (2008; 7/89, 7.87%). That our sample group was composed of teachers in a continuing education program may explain this result.

Unexpectedly, “career transition and changes,” a frequently cited CWB component in Kidd (2008), who highlighted its difference from the components discussed in mainstream well-being perspectives, was not frequently mentioned by the participants of our study. This may be a result of the characteristics of a career in teaching, which is considered relatively stable and is among the most popular occupations in Taiwan. A mix of both positive and negative affectivity at various phases of career transition (Kidd, 2008) may explain career transition being reported by only 11 participants (8.15%). Asking the participants questions focused on negative affectivity may also have resulted in this contrasting result. A period of wellness perceived during careers was reported as eliciting “a sense of purpose and direction for the future” (12/135, 8.89%), which indicated the pursuit of personal goals throughout careers.

“Work–life balance” and “autonomy at work” were seldom reported by our participants. These CWB components were present in 5.93% of the statements. This result was consistent with Kidd’s (2008) findings. Despite the dominant role of work in an individual’s life, autonomy or sufficient time left for leisure activities and family life were crucial to eight participants’ sense of CWB.

Only two participants (Participants 93 and 134) reported that “participation in decision-making” made them feel positive about their careers. This may be due the lack of managerial positions held by most participants in this study, and these young participants had relatively little experience in working in current schools (their average organizational tenure was less than 6 years).

#### Conceptualization: Applying the self-determination theory to career well-being components

Following Ryan and Deci’s (2000) argument regarding the importance of needs satisfaction, we reorganized the CWB components to ensure they corresponded to the needs suggested in the SDT. As Chen (2017) argued in his study of CSDT, competence, relatedness, and autonomy can be conceptualized as aspects of the agency that determines career wellness as well as work-related well-being, as discussed by Deci et al. (2017). Consequently, our conceptualization of CWB was established. In Table 2, three basic psychological needs are linked to CWB components. Factor 1 explains how wellness is caused by the “person-environment fit” (PEF). With respect to satisfying the need for competency, “work performance and achievement” is the primary source of CWB. According to our results, the need for relatedness was largely satisfied by “the interpersonal relationships and support at work,” which was examined in Factor 2, (interpersonal support, IS). In Factor 3, “career balance” (CB) was composed of “autonomy at work” and “work–life balance,” which are both related to the need for autonomy. As Ryan and Deci (2001) suggested, satisfying psychological needs contributes to a happy life where positive affectivity is experienced strongly as a self-determined agent of happiness. These factors satisfying the needs for competence, relatedness, and autonomy could be considered the necessary working conditions that elicit a state of well-being in which basic psychological needs are satisfied. Louis (1998), the teaching profession’s pioneer in teacher retention strategy and school effectiveness, determined that the achievement of this wellness provides a fundamental basis for teachers’ professional development.

**TABLE 2:** A conceptualization of CWB.

Psychological needs	Theory-relevant factors	Components	Descriptions
Basic psychological needs	**CWB Factor 1:** **Person-environment fit (PEF)** Relevant to the satisfaction of a need for competency	Work performance and achievement (Component 2)	This factor described the person-environment (PE) fit by which wellness was experienced through professional competence. It referred to the wellness experienced by performing one’s own professional tasks with competence, an interest in the scheduled tasks, and having a clear target or direction for continued advancement in work performance. The sense of achievement that caused teachers to feel positive about careers, including getting along with students and their parents.
	**CWB Factor 2:** **Interpersonal support (IS)** Relevant to the satisfaction of a need for social relatedness	Interpersonal relationships and support at work (Component 3)	This factor described the wellness associated with work support. It referred to the wellness experienced by working in a friendly workplace, and receiving support, feedback and recognition from others. This involved getting along with the school supervisor, administrative staff, and colleagues, and provided teachers with a source of CWB.
	**CWB Factor 3:** **Career balance (CB)** Relevant to the satisfaction of a need for autonomy	Autonomy at work (Component 7) Work–life balance (Component 6)	This factor described the wellness associated with career balance. It referred to the wellness experienced by perceiving a high level of autonomy at work. Home life or leisure time was not affected by work, thus demonstrating a balance between work and family life.
Career growth needs	**CWB Factor 4:** **Proactive career management (PCM)** Relevant to the satisfaction of career fulfillment needs	Career transition and changes (Component 4) A sense of purpose and direction for the future (Component 5) Self-development and learning (Component 1) Participation in decision-making (Component 8)	This factor described the wellness associated with proactive career behaviors (PCB). It referred to the wellness experienced by career changes (holding a new position, the establishment of a new career pattern, or the extension of one’s social network and living community due to a new job). Well-being was strengthened by several approaches to pursuing self-development and learning (participated in training and programs to learn new knowledge, skills, or professional development; obtained learning opportunities through advancing in a current job; or received a diploma). Having participated in important decisions also allowed some teachers to exercise control over the work environments in which they developed their careers and also led to a sense of wellness. Seeing future prospects for development represented the wellness associated with the PCB for the pursuit of clear targets for career goals.
			

In addition to these three factors that are directly related to the well-being experienced due to the satisfaction of psychological needs, Factor 4—proactive career management (PCM)—was used to analyze the remaining CWB components (career transition and changes, a sense of purpose and direction for the future, self-development and learning, participation in decision-making). This CWB factor examined engagement in proactive career management and the self-directed tendency to remove career barriers or seek career opportunities, rather than in-role work behaviors. The various wellness outcomes due to responsive and proactive career behaviors have been discussed in recent career management literature (Hirschi, 2014). Given the specific characteristics of PCM, PCM is likely to be facilitated by additional mechanisms addressed in the PWB literature—chiefly in the eudaimonic tradition (Ryff and Keyes, 1995) rather than the hedonic tradition. However, this discussion is beyond the scope of our study.

## Study 2: Psychometric examination of career well-being

In Study 1, we conceptualized CWB by employing SDT to elucidate the components of CWB. In Study 2, we quantitatively analyzed this conceptualization by using EFA.

### Method

#### Respondents and questionnaire

A survey was administered to 191 teachers in the south of Taiwan. Their mean age was 38.09 years [standard deviation (SD) = 11.06]. Approximately 60% were married, and 30% were men. Nearly half of them reported following no religion. The mean number of years of teaching experience was 11.97 (SD = 7.74), and the mean organizational tenure was 7.77 years (SD = 6.81). This indicates that the study sample was composed predominantly of senior teachers.

The results obtained in Study 1 informed the design of the questionnaire used for Study 2. This questionnaire was used to assess the respondents’ CWB experiences, needs satisfaction, and several work-related variables. The respondents were then asked about their backgrounds, including age, marital status, gender, religion, teaching experience, and organizational tenure.

#### Development of a career well-being measurement instrument

To measure CWB, the respondents were asked to reflect on their working life and to identify the most appropriate response to 16 items corresponding to the four CWB factors. The items were devised on the basis of the results of Study 1 and were expressed in the form of statements about personal feelings of pleasure that lasted throughout career development (see Appendix 1). Before the data collection, the items were reviewed by five school teachers, who provided their opinions and made suggestions for modifications in the wording of the statements to ensure they were appropriate to the current teachers. In addition to using expert judges (Hardesty and Bearden, 2004), we used item analysis and evaluations of the reliability and validity of the CWB measurement scale to identify an optimal solution by reducing the number of items (Moses, 2017; Luo et al., 2019; Schumacker, 2019). Finally, the participants’ responses on the items were calculated in terms of the four CWB factors: PEF (three items), IS (four items), CB (three items), and PCM (six items).

#### Additional instruments

Four instruments were also included in the questionnaire to determine the validity of the CWB components. A Chinese version of the Basic Psychological Need Satisfaction Scale available in a cross-cultural study (Chen et al., 2015) was employed to assess the satisfaction perceived related to competence (three items, e.g., Cronbach’s alpha = 0.79), relatedness (three items, Cronbach’s alpha = 0.72), and autonomy (three items, Cronbach’s alpha = 0.47). The measurement equivalence and invariance of this model across four countries was confirmed, demonstrating that this scale was appropriate for used in Chinese cultural settings.

In addition to this instrument for analyzing theoretical validity, other instruments were employed to examine the criterion-related validity. The Teacher Self-Efficacy Scale devised by Vieluf et al.’s (2013) was used to assess teachers’ self-efficacy. This instrument was previously used in the Organization for Economic Co-operation and Development (OECD) Teaching and Learning International Survey for a cross-national comparison of teachers’ self-efficacy among 23 countries. The construct validity and reliability of this scale was confirmed (four items, Cronbach’s alpha = 0.82). Additionally, the Colleague Support Scale was adapted to the Multidimensional Scale of Perceived Social Support (Zimet et al., 1988). Its high level of measurement quality was supported in a study of Chinese culture (Chou, 2000). The four items described the support received from work colleagues (Cronbach’s alpha = 0.89). The final instrument employed in this study was the Chinese version of the Career Autonomy Scale, which demonstrated the two-factor structure of work autonomy and life autonomy. This scale was originally established with special reference to the description of autonomy noted in the Job Diagnostic Survey (Hackman and Oldham, 1975). Its construct validity was confirmed by using exploratory factor analysis (Lin and Yang, 2012). The three items that were relevant to work autonomy were used in this study to assess the extent to which a worker’s job provided them with freedom and independence and how they were involved with the procedures of scheduling and completing their work (Cronbach’s alpha = 0.86).

These scales used in this study were converted to a four-point Likert scale format to maintain consistency, with response options ranging from “strongly disagree” to “strongly agree.”

#### Procedure

Printed questionnaires were delivered to prospective respondents. They were informed of the purpose of this study and were assured of the confidentiality of their responses. Participation in the study was voluntary. The respondents who returned the completed questionnaires and shared their email address were eligible for a raffle for 15 USB flash drives. After data collection, we analyzed the data using R packages for item analysis and EFA (Luo et al., 2019; Schumacker, 2019).

### Results

#### Descriptive statistics

Descriptive statistics for key measures are reported in Table 3. The means for the CWB factors were between 2.42 and 3.04 (SD = 0.48–0.54). Almost no means for the additional variables were outside the range of 3 and 3.14 (SD = 0.42–0.52). The means of the CWB factors were slightly lower than the other variables; in particular, PCM was the lowest (mean = 2.42, SD = 0.53).

**TABLE 3:** Descriptive statistics and correlations of the variables.

	1	2	3	4	5	6	7	8	9	10
1. CWB 1: PEF	3.04 (0.48)									
2. CWB 2: IS	0.53**	3.00 (0.54)								
3. CWB 3: CB	0.49**	0.54**	2.97 (0.54)							
4. CWB 4: PCM	0.36**	0.34**	0.31**	2.42 (0.53)						
5. CNS	0.55**	0.43**	0.32**	0.31**	3.06 (0.46)					
6. RNS	0.38**	0.40**	0.23**	−0.01	0.51**	3.13 (0.44)				
7. ANS	0.62**	0.38**	0.28**	0.28**	0.62**	0.58**	2.98 (0.42)			
8. TSE	0.45**	0.33**	0.26**	0.18*	0.47**	0.37**	0.41**	3.04 (0.44)		
9. CS	0.34**	0.38**	0.28**	−0.09	0.16*	0.50**	0.39**	0.34**	3.14 (0.52)	
10. WA	0.44**	0.38**	0.39**	−0.02	0.32**	0.36**	0.47**	0.38**	0.46**	3.07 (0.45)

*n* = 191. *p < 0.05, **p < 0.01.

CWB 1: PEF, person-environment fit (work performance and achievement); CWB 2: IS, interpersonal support (interpersonal relationships and support at work); CWB 3: CB, career balance (autonomy at work, work–life balance); CWB4: PCM, proactive career management (a sense of purpose and direction for the future, self-development and learning, career transition and changes, and the participation in decision-making); CNS, competence need satisfaction; RNS, relatedness need satisfaction; ANS, autonomy need satisfaction; TSE, teacher self-efficacy; CS, colleague support; WA, work autonomy.

#### Item analysis and reliability of measurement scale

In addition to expert analysis, our item analysis involved statistics used to evaluate the quality of individual items on the scale as well as the relationships among the items. The high–low 27 percent group method (Fan, 1954; Moses, 2017) was employed to determine the item discrimination of the CWB scale. The results indicated that the item-total correlation values were high (correlation coefficients ranged between 0.39 and 0.63) and the differences between the high and low 27 percent groups were significant at the *p* < 0.001 level (Table 4). Therefore, the 16 items were retained and classified into four CWB factor groups: PEF (three items), IS (four items), CB (three items), and PCM (six items).

**TABLE 4:** Item analysis results.

	Low 27 percent group Mean	High 27 percent group Mean	Discrimination	CR value	*P*-Value	Item-total correlation	Item-subscale correlation	Cronbach’s alpha if item deleted	Subscale score reliability if item deleted
PEF1	2.78	3.50	0.72	6.90	0.00	0.52	0.67	0.87	0.71
PEF2	2.73	3.48	0.75	6.86	0.00	0.53	0.75	0.87	0.62
PEF3	2.51	3.44	0.93	8.66	0.00	0.59	0.54	0.87	0.85
IS1	2.73	3.50	0.77	6.83	0.00	0.52	0.70	0.87	0.73
IS2	2.80	3.50	0.70	6.79	0.00	0.47	0.64	0.87	0.77
IS3	2.33	3.33	1.00	8.22	0.00	0.57	0.61	0.87	0.78
IS4	2.42	3.52	1.10	9.18	0.00	0.63	0.61	0.87	0.78
CB1	2.55	3.38	0.84	7.87	0.00	0.54	0.50	0.87	0.84
CB2	2.45	3.44	0.99	8.44	0.00	0.51	0.73	0.87	0.60
CB3	2.53	3.46	0.93	7.96	0.00	0.54	0.67	0.87	0.66
PCM1	1.95	2.92	0.98	8.81	0.00	0.56	0.65	0.87	0.80
PCM2	1.78	2.77	0.99	6.83	0.00	0.46	0.64	0.87	0.81
PCM3	2.35	3.08	0.73	6.28	0.00	0.39	0.48	0.88	0.84
PCM4	2.04	3.06	1.02	8.48	0.00	0.56	0.61	0.87	0.81
PCM5	1.96	2.92	0.96	7.88	0.00	0.51	0.62	0.87	0.81
PCM6	1.95	2.81	0.86	6.82	0.00	0.52	0.68	0.87	0.80

*n* = 191; PEF, person-environment fit (work performance and achievement); IS, interpersonal support (interpersonal relationships and support at work); CB, career balance (autonomy at work and work–life balance); PCM, proactive career management (a sense of purpose and direction for the future, self-development and learning, career transition and changes, and the participation in decision-making).

Cronbach’s coefficient α was used to calculate the internal consistency of the CWB scale. The results indicated that the scale had an acceptably high value (α = 0.88). In addition, the items for each factor were acceptable within the factor (α = 0.80 for PEF; α = 0.81 for IS; α = 0.79 for CB; α = 0.84 for PCM). The α coefficients verified that the overall reliability of the CWB measure was acceptable.

#### Validity examinations of the career well-being measure: Exploratory factor analysis results

Exploratory factor analysis was employed as a data analysis strategy to explore the factor structure of CWB. EFA was conducted after item analyses by using an R package (i.e., psych version 2.2.5). The factorability of the matrix was determined using the Kaiser–Meyer–Olkin Measure of Sampling Adequacy (overall measure of sampling adequacy = 0.83) and Bartlett’s test of sphericity (χ^2^ = 1423.316, *p* < 0.001). We followed the recommendation of Barendse et al. (2015) of obtaining polychoric correlations by using weighted least squares estimation to determine the dimensionality of discrete responses (i.e., a four-point Likert scale format) because the recommendation is theoretically justified, and the method leads to fewer convergence problems than other estimation methods. Oblimin rotation was used to transform the vectors associated with the factor analysis into a simple structure.

The results revealed that “PCM,” “IS,” “PEF,” and “CB” were loaded on four dimensions. All items loaded on the expected dimensions. The four-factor solution appeared the best representation of the latent structure of the CWB scale because all items had high communalities. The majority of the items consistently loaded on one factor. Only one item (i.e., PEF3) did not (factor loadings greater than 0.3). The final EFA results are listed in Table 5.

**TABLE 5:** EFA results.

	Factor loadings				
Items	PCM	IS	PEF	CB	*h* ^2^
PCM6	0.77	−0.12	0.01	0.12	0.60
PCM2	0.73	−0.04	0.07	−0.10	0.52
PCM1	0.72	0.09	0.05	−0.08	0.56
PCM5	0.66	−0.07	−0.06	0.24	0.51
PCM4	0.62	0.18	−0.09	0.09	0.48
PCM3	0.51	0.21	0.03	−0.24	0.34
IS1	−0.08	0.78	−0.05	0.15	0.66
IS2	−0.12	0.71	0.11	0.00	0.56
IS3	0.20	0.61	0.10	−0.10	0.51
IS4	0.19	0.60	0.05	0.09	0.54
PEF2	−0.05	−0.02	0.99	0.04	0.98
PEF1	0.08	0.05	0.70	0.02	0.58
PEF3	0.32	0.19	0.43	−0.04	0.47
CB2	0.02	0.01	0.10	0.84	0.79
CB3	0.04	0.17	0.02	0.70	0.63
CB1	0.12	0.28	0.12	0.35	0.41

*n* = 191; PEF, person-environment fit (work performance and achievement); IS, interpersonal support (interpersonal relationships and support at work); CB, career balance (autonomy at work and work–life balance); PCM, proactive career management (a sense of purpose and direction for the future, self-development and learning, career transition and changes, and the participation in decision-making).

In addition to the convergent and divergent validity, the validity of the CWB measurement was examined, in terms of the relationships of the four CWB factors with the three needs of autonomy, relatedness, and competence (for examining the theoretical validity), as well as with the teacher’s self-efficacy, colleague support, and work autonomy (the criterion-related validity).

Generally speaking, the divergent and convergent validity was supported by the results of the correlation analyses (see Table 3). The Pearson correlation coefficients of the four CWB factors ranged between 0.31 and 0.54 and were deemed to be significant at the *p* < 0.01 level. Significant correlations were also observed between the CWB factors (PEF, IS, CB, PCM) and CNS, RNS, ANS, with the Pearson correlation coefficients ranging between 0.23 and 0.62. Although the relationship between the fourth CWB factor (PCM) and RNS was not significant (*r* = −0.01), its correlations with CNS and ANS (*r* = 0.31, 0.28, respectively) both achieved statistical significance. Overall, the validity of this theory-based measurement was supported.

By closely examining the criteria, we determined that the relationships that were expected to occur between PEF and teachers’ self-efficacy (*r* = 0.45), IS and colleague support (*r* = 0.38), and CB and work autonomy (*r* = 0.39), all achieved statistical significance at the same level (*p* < 0.01). In particular, PEF, IS, and CB were revealed to exhibit the highest Pearson correlation coefficients among their relationships with their corresponding criterion (namely teacher self-efficacy for PEF, colleague support for IS, and work autonomy for CB). Accordingly, we reached the conclusion that the measurement that was established in our conceptualization in Study 1 (the four-factored structure of CWB), achieved a high level of criterion-related validity. In addition, the slightly lower degrees of correlation (*r* = 0.45, 0.38, 0.39), in contrast to the correlations among the first three CWB factors, also provided divergent and convergent validity. This result demonstrated that the nature of CWB was conceptually different from those work-related variables.

## Study 3: Psychometric verification of the career well-being scale

In Study 3, we employed CFA as a data analysis strategy to verify the factor structure of CWB. To establish an optimal balance between goodness-of-fit and parsimony as Marsh et al. (2013, 2020) suggested, ESEM was employed. This enabled us to broaden our understanding of the organization of CWB. Researchers have reported that when using simulated data (e.g., Xiao et al., 2019; Wei et al., 2022) and constructing scales (e.g., Fresno et al., 2020; Ng et al., 2017; Gegenfurtner and Quesada-Pallarès, 2022), ESEM can offer key information. In this study, we used ESEM to determine the latent optimal structure of the CWB model established in our previous two studies. This modeling approach can be useful for estimating models with potentially unknown cross-loadings (Wei et al., 2022). We therefore strengthened our CWB measure by applying two estimation approaches (i.e., CFA and ESEM).

### Method

#### Respondents

A total of 533 teachers participated in this study. The mean age was 35.03 years (SD = 8.37). Approximately 40% were married, and 23% were men. Nearly half reported having no religious beliefs. The mean number of years of teaching experience was 8.88 (SD = 6.9), and the mean organizational tenure was 5.53 years (SD = 5.96). This information indicates that the study sample mainly comprised senior teachers, which was similar to the sample obtained in the previous study.

#### Confirmatory factor analysis results

Confirmatory factor analysis with Mplus was employed to verify the CWB factor structure. Several indices were used to assess the goodness-of-fit of the model, including χ^2^*/df*, the comparative fit index (CFI), root mean square error of approximation (RMSEA), and standardized root mean square residual (SRMR).

The bootstrapping method was employed (Bootstrap = 5000), and the preliminary CFA results revealed that the four-factored model established in Study 2 was mildly supported (χ^2^ = 666.36, *df* = 98, χ^2^*/df* = 6.80, CFI = 0.87, RMSEA = 0.10, SRMR = 0.09). The information obtained through the goodness-of-fit test indicated that ESEM was required to broaden our understanding of the organization of CWB (Marsh et al., 2013; Fresno et al., 2020; Dierendonck et al., 2021; Gegenfurtner and Quesada-Pallarès, 2022).

#### Exploratory structural equation modeling results

Exploratory structural equation modeling with Mplus can be used to analyze models with potentially unknown cross-loadings. SEM researchers (e.g., Fadda et al., 2020) suggested that ESEM integrates the strengths of EFA and CFA and, therefore, is useful for scale validation. Substantial cross-loadings violate the CFA assumption of zero magnitude cross-loadings (i.e., simple structure). Therefore, in cases with such cross-loadings, ESEM should be used to assess the internal structure of multidimensional constructs (Ng et al., 2017).

The most favorable fit indices were obtained for the ESEM solution (χ^2^ = 229.05, *df* = 62, χ^2/^*df* = 3.69, CFI = 0.96, RMSEA = 0.07, SRMR = 0.03), demonstrating its superiority over the CFA model. The ESEM results revealed that cross-loadings required consideration in the analyses of the latent structure underlying the CWB scale (Table 6). Although CFA is frequently considered the most favorable model for validating internal structure, our ESEM results demonstrate that small cross-loadings of factor indicators can more realistically represent respondent data. The ESEM results revealed that all of the items had substantial loadings on their respective factors except IS4, and one item (i.e., PCM3) had slightly high factor loadings on more than one factor (greater than 0.3). We determined that CFA was not well suited for CWB data, as Fadda et al. (2017, 2020) also indicated in their recent well-being studies.

**TABLE 6:** CFA and ESEM results.

		CFA				ESEM			

Factor loadings		PEF	IS	CB	PCM	PEF	IS	CB	PCM
Person-environment fit (PEF)	PEF1	0.85	0.00	0.00	0.00	0.90	−0.01	−0.01	−0.04
	PEF2	0.85	0.00	0.00	0.00	0.86	0.04	−0.09	0.01
	PEF3	0.60	0.00	0.00	0.00	0.33	0.09	0.16	0.27
Interpersonal support (IS)	IS1	0.00	0.80	0.00	0.00	0.11	0.74	0.09	−0.08
	IS2	0.00	0.75	0.00	0.00	0.01	0.85	−0.02	−0.06
	IS3	0.00	0.71	0.00	0.00	−0.07	0.64	0.01	0.24
	IS4	0.00	0.66	0.00	0.00	0.24	0.18	0.36	0.10
Career balance (CB)	CB1	0.00	0.00	0.86	0.00	−0.07	0.01	0.90	0.01
	CB2	0.00	0.00	0.86	0.00	−0.02	0.02	0.85	0.02
	CB3	0.00	0.00	0.70	0.00	0.14	0.18	0.51	0.04
Proactive career management (PCM)	PCM1	0.00	0.00	0.00	0.71	0.05	0.08	−0.04	0.65
	PCM2	0.00	0.00	0.00	0.81	−0.06	−0.06	−0.03	0.92
	PCM3	0.00	0.00	0.00	0.65	−0.02	0.35	−0.03	0.49
	PCM4	0.00	0.00	0.00	0.75	−0.03	−0.12	0.02	0.83
	PCM5	0.00	0.00	0.00	0.62	0.08	−0.08	0.15	0.53
	PCM6	0.00	0.00	0.00	0.56	0.17	0.04	−0.03	0.46
PEF		–				–			
IS		0.70	–			0.60	–		
CB		0.46	0.66	–		0.45	0.51	–	
PCM		0.50	0.59	0.59	–	0.46	0.48	0.55	–

*n* = 533; PEF, person-environment fit (work performance and achievement); IS, interpersonal support (interpersonal relationships and support at work); CB, career balance (autonomy at work and work–life balance); PCM, proactive career management (a sense of purpose and direction for the future, self-development and learning, career transition and changes, and the participation in decision-making).

## Discussion

### General discussion

A new conceptualization of CWB was established, and SDT was employed to construct a four-factored structure to explain the components categorized in this study. Since Kidd’s (2008) model, few studies have attempted to further conceptualize CWB. Additionally, limited studies have attempted to establish a practical method of measuring not only career practices, but also how CWB measurements can be employed for continuous investigations into workers’ well-being experience throughout the course of their working life. Our results provide another approach complementary to the CWB conceptualization that was documented in Kidd (2008), demonstrating the common features shared between Eastern and Western cultures as well as the existence of some variations that depend on cultural and occupational contexts. Although our conceptualization was established in Taiwan, the characteristics of CWB experienced by teachers can still be documented in terms of three aspects, which are discussed as follows.

First, CWB is an umbrella concept for a range of wellness attributes that workers perceive in relation to their careers. The primary three CWB factors that correspond to the psychological needs for competence, relatedness, and autonomy demonstrate positive affectivity that is ordinarily caused by the satisfaction of basic needs. Through needs satisfaction, a sense of wellness can be strengthened and perceived over the long-term. This result supports the emphasis on SDT regarding basic psychological needs and the pursuit of happiness (Ryan and Deci, 2000, 2001). These common components that contribute to affective experience and exist among different types of well-being are applicable in the context where needs for competence, relatedness, and autonomy are satisfied. CWB experienced through ongoing career development actions and activities is also included. As Diener et al. (2018) recommended, SWB cannot be specified clearly without clarifying the rationale underlying the fundamental optimal function that determines the fulfillment of fundamental psychological needs. In CWB research, taking into account these three basic psychological needs helps to establish a theory-based CWB measurement and strengthens the theoretical ground through an emphasis on the human agency that explains affective experience throughout an individual’s long-term career (Hartung, 2011; Chen, 2017). Our study provides a preliminary conceptualization and evidence for further verifications.

Second, as we expected, the sense of wellness that workers experience throughout careers exhibited features that were different from those of SWB that have been widely analyzed in the literature. Further, our results demonstrated that the career experiences that created a perception of wellness throughout careers are somewhat different from those found in Western studies. In addition to the satisfaction of basic psychological needs, PCM demonstrated distinguishing features that determined how CWB is different from the traditional definition of SWB (e.g., a higher positive affect). Although a similar result was revealed in Kidd (2008), our study specified this feature of CWB much more clearly and explained this difference in terms of the importance of satisfying career growth needs for teachers’ general sense of wellness experienced throughout their lives and in their career choices and development. As Higgins et al. (1997) stated, emotional responses (emotional frequency and intensity) to goal attainment vary, depending on the type of goal. A distinction between the ideal goals and ought goals explains the different levels of wellness that were both included in our CWB conceptualization and in that of Kidd (2008). Whereas the ought goals (duties and responsibilities) are achieved to generate the wellness with a preventative focus, the chronic ideal goals (hopes and aspirations) facilitate the overall promotion of wellness. Obviously, PEF, IS and CB satisfy the psychological need for competence, relatedness, and autonomy through meeting the requirements necessary for doing a job successfully. The importance of three basic psychological needs has been explained much more clearly in the CSDT of Chen (2017), which specifies the rationale underlying the ongoing sense of workers’ well-being that persists throughout their career activities and actions. However, the promotion of wellness over a period of time can be enhanced further by the pursuit of hopes and aspirations. Such ideal goals are likely to be demonstrated in terms of PCM.

Third, PCM featured in this study echoed the importance of PWB (Ryff, 1989) and there has been a growing argument for PCM (Hirschi, 2014) regarding the wellness experienced within contemporary careers. As conceptualized in the CSDT of Chen (2017), the pursuit of wellness that is associated with engagement with and the meaning of long-term goals throughout careers is a distinctive trait of CWB. From this point of view, subjective meanings ascribed and meaning-making processes undertaken when constructing a career elicit a sense of career wellness. Notably, the combination of a personal striving for success with wellness did not clearly distinguish this study from Kidd (2008). Similar to British workers who emphasized learning and development, sense of purpose, and their relationships with an organization (e.g., exercising personal control and power over their work environment) and career transitions (voluntarily moving into a new role or career pattern) (Kidd, 2008), Taiwanese teachers who participated in this study demonstrated high levels of interests in PCM and also benefited from those career experiences (self-development and learning, a sense of purpose and direction for the future, the participation in decision-making, and career transition and changes).

Scale development and validation are critical aspects of CWB research. Statistical analyses verified the convergent, divergent, and criterion validity of our CWB measurement model. Although small cross-loadings were detected, the items of the measure had substantial loadings on their respective factors. Furthermore, the less restrictive assumptions of ESEM offered more favorable model and data fit than the CFA model did. As Ng et al. (2017) reported, the allowance of small cross-loadings of ESEM accounts for imperfect indicators and social desirability responding. The results obtained in this study are consistent with others regarding the employment of ESEM for scale development and verification (e.g., Marsh et al., 2013, 2020; Fadda et al., 2017, 2020; Ng et al., 2017; Luo et al., 2019; Xiao et al., 2019; Wei et al., 2022). This modeling approach, which integrates the strengths of EFA and CFA, may be a candidate for further evaluation.

### Limitations and implications for future study

The need-satisfaction theoretical model (Ryan and Deci, 2000) was discussed in relation to the components categorized in our results, resulting in the development of a four-factor structure of CWB. The first three factors (PEF, IS, and CB) were linked to basic psychological needs, but the link between PCM and the satisfaction of psychological needs remains unclear. Further investigations are required to clarify the exact relationships.

The workers’ CWB experiences associated with negative affectivity were not investigated in this study, as they were closely related to “meaning constructs” (e.g., searching for meaning) which frequent mix of both positive and negative affectivity (Kidd, 2008; Proctor et al., 2015). However, the PWB model examined by Ryff (1989) demonstrated a competitive advantage provided by the eudaimonic tradition in finding the fundamental sense of meaning that is especially linked to well-being. Although the meaningfulness associated with well-being was not the main focus of our study, future studies should examine how wellness can be created in these negative events through cognitive changes in the meaning or purpose of careers (Ryff, 1989). Chen (2017) attempted to incorporate meaning-making mechanisms into the study of CWB. Beyond the existing approach to elicit crucial career experiences with positive affectivity, future research should not only focus on career events with negative affectivity but also explore the fundamental mechanisms of affectivity to facilitate the advancement of CWB theory.

Notably, different from the three CWB components that describe the results of establishing a better fit between personal attributes and the characteristics of work environments in the creation of wellness, proactive attitudes and the behaviors that are performed in advance in sensing and initiating opportunities for strengthening career enhancement (i.e., PCM) would contribute to the long-term satisfaction of psychological needs (Tims et al., 2016). This CWB factor demonstrates an exact fit with the existing research in terms of long-term wellness in careers. CWB, as described in light of proactive strategies, entails an advanced level of psychological functioning to meet career growth needs, as addressed by Ryff and Keyes (1995) in the PWB model. Longitudinal well-being, namely the pursuit of happiness through goal attainment and fulfillment, has also been discussed through the self-concordance model (Sheldon and Elliot, 1999) that stresses the importance of a healthy relationship with goals. The PCM highlights how career opportunities can be created out of the long-term pursuit of purpose and goal striving and can further lead to a state of well-being that is qualitatively different from the wellness associated with the majority of significant career events investigated in our studies. Further investigations focusing on PCM are required to advance CWB theory and practice.

Several methodological issues may have affected this study. Our sample selection method, which entailed collecting data from a continuing education program, may have biased our results. A distortion caused by sample characteristics that our sample is not representative of the teacher population in Taiwan may compromise the results. According to Education Statistical Indicators (2021), the percentage of female teachers at primary and secondary schools is 72.06% and 63.60%, respectively. The percentage of female teachers who participated in our studies was slightly higher (70–77%). This limitation substantially limits the applicability of our findings for a large population of school teachers.

The well-being model established in our studies is not equally applicable to all occupations. Teachers are driven by needs that may differ considerably from those of individuals in other professions. Their psychological needs, such as developing relationships with students and feeling competent and autonomous, may be easier to fulfill. Furthermore, teachers are infrequently motivated by money or power. Approximately 70% of the participants of this study were women; this may indicate that many chose this profession under the assumption that they would earn a second income for their household. This would enable them to engage in a profession, which can be fulfilling, rather than focusing on meeting the needs of their families. For these reasons, teachers may be a biased sample set, which could prevent our results from being used to establish a comprehensive understanding of CWB. The narrowness of our sample set considerably limits the applicability of our studies and prevents our findings from being generalizable to other occupations. Furthermore, because our sample solely comprised Taiwanese teachers, determining whether differences in our findings and those of other applications of CWB occurred because of differences between cultures or differences between teachers and those in other professions is impossible.

Due to the cross-sectional design of this study, we cannot clarify the potential causality between CWB factors and the three basic psychological needs. To ensure construct validity and reliability, CWB data should be collected consistently from a large and representative sample of a target population. Furthermore, a longitudinal design must be employed to establish the longitudinal factorial construct validity, internal consistency, and temporal stability of the CWB scale and to evaluate its consistency over time (Morgado et al., 2017; Van Zyl et al., 2021). In addition to the cross-sectional construct validity, which was evaluated in our studies, the longitudinal validity of the CWB scale should be evaluated by investigating prespecified hypotheses regarding the associations of the main factors of the scale with other measures.

Although the measurement model was established on the basis of a conceptualization that incorporates SDT into CWB factors, additional equivalent models may be available that also statistically fit the data. In consideration of convenience and the flexibility of using first-order models in measurement practice, we concentrated on the development of the “first-order four-factor model” in this study. However, high-order models may be specially applicable; this must be explored, clarified, and confirmed by future research.

As noted in Study 3, ESEM uses the favorable aspects of EFA and CFA and offers advantages over the traditional CFA and EFA approaches. ESEM offers a robust, rigorous, and flexible analytical means of estimating a less restrictive measurement model that permits cross-loadings, leading to useful fit indices and parameter estimates. ESEM can be used to simultaneously fit the CFA and EFA models and, therefore, is more suited for theoretical conceptualization of constructs and measurement instruments (Van Zyl and Ten Klooster, 2021). Marsh et al. (2020) argued this is the main reason that ESEM has been increasingly employed as a data analysis strategy for verifying the factor structures of scales. Employing this approach to determine how well factorial structures fit CWB data (i.e., measurement models) and to explore and analyze the structural associations between the main CWB factors and other measured variables and latent constructs (i.e., structural models in SEM) warrants further investigation.

Asking questions that required participants to reflect on the factors they considered most crucial to achieving or having high CWB may have been a more accurate means of data collection. However, determining whether an experience exemplified high or low CWB is impossible. Participants may have never had a high-CWB experience or their CWB experience may have been missing key factors because they had not yet encountered them. Therefore, establishing and verifying an alternative approach to CWB measurement to that used in our studies is necessary to advance CWB theory.

Further cross-cultural research should be conducted, with a robust theoretical framework used to investigate CWB from a cultural perspective. Due to the lack of a cross-cultural study design, cultural differences were not clearly detected in this study. However, Lu and Gilmour (2004) noted the distinct characteristics between individually oriented well-being (personal accountability and explicit pursuit) and socially oriented well-being (role obligation and dialectical balance). Future studies should continue this line of investigation, to clarify how CWB varies between cultures.

Finally, regarding a further examination of CWB, career satisfaction should be taken into account to examine its relationship with the CWB factors that have been revealed in our study. Proctor et al. (2015) suggested that satisfaction variables (e.g., life satisfaction) should be considered as a consequence of SWB or PWB. It is therefore not appropriate to include satisfaction in the measurement of well-being before the direction of causality between satisfaction and well-being variables has been examined clearly. This argument should not be examined independently without using longitudinal study designs.

## Conclusion

Our conceptualization of a four-factor structure was supported through a statistical analysis. A theory-based and psychometrically sound measurement model was established in this study for evaluating the wellness experienced throughout teaching careers. This measurement model can be used for the regular evaluation of teachers’ CWB, and also in the in-service training and counseling practices provided to them. Further researchers should focus on negative career events and link the rationale that underlies SDT to explain workers’ long-term perceptions of their experiences of meaningfulness at work. Determining how these career events can be generated and contribute to proactive career management in various occupations and cultures can advance our knowledge of CWB.

## Data availability statement

The data used in this study will be made available upon reasonable request. Further inquiries can be directed to the corresponding author, PY, p.yang71@yahoo.com.

## Ethics statement

Ethical review and approval was not required for the study on human participants in accordance with the local legislation and institutional requirements. The patients/participants provided their written informed consent to participate in this study.

## Author contributions

M-NY and PY conceived of the presented idea. PY developed the theory and verified the analytical methods. M-NY encouraged PY to investigate career well-being and gave advice on the findings of his work. Both authors discussed the results and contributed to the final manuscript. Both authors contributed to the article and approved the submitted version.

## Appendix 1

### Career well-being scale

Please reflect on your working life and identify the most appropriate responses to the following items that correspond to the source of your career well-being.

Please rate each item from 1 (strongly disagree) to 4 (strongly agree) to indicate the degree to which you agree with each item; responses should be based on your personal feelings of pleasure that have come from and persisted throughout your teaching career (“1” refers to strongly disagree; “4” refers to strongly agree).

1. Performing to the best of my ability
2. Personal interests in line with scheduled tasks
3. Worked in a friendly workplace
4. Got along with colleagues
5. Got along with school supervisor and administrative staff
6. Participated in important decisions
7. Had a clear target or direction for continued advancements in work performance
8. Professional development in education
9. Participated in on-the-job (OJT) training and development programs
10. Saw prospects for future development
11. Expanded my social network and living community due to a new job
12. Held a new position or established a new career pattern
13. Perceived autonomy at work
14. Home life and leisure time were not affected by work
15. Achieved a balance between work and family life
16. Received recognition from others

To make a scale-purification decision, the items marked in gray were removed from the scale.
